# Time-Series Transcriptome Analysis Reveals the Molecular Mechanism of Ethylene Reducing Cold Sensitivity of Postharvest ‘Huangguan’ Pear

**DOI:** 10.3390/ijms24065326

**Published:** 2023-03-10

**Authors:** Chuangqi Wei, Yanyan Wu, Zhenyu Ma, Yudou Cheng, Yeqing Guan, Yang Zhang, Yunxiao Feng, Xueling Li, Junfeng Guan

**Affiliations:** 1 Institute of Biotechnology and Food Science, Hebei Academy of Agriculture and Forestry Sciences, Shijiazhuang 050051, China; 2 Plant Genetic Engineering Center of Hebei Province, Shijiazhuang 050051, China

**Keywords:** pear, cold sensitivity, RNA-seq, WGCNA, WRKY31

## Abstract

‘Huangguan’ pear (*Pyrus bretschneideri* Rehd) fruit is susceptible to cold, characterized by developing peel browning spots (PBS) during cold storage. Additionally, ethylene pretreatment reduces chilling injury (CI) and inhibits PBS occurrence, but the mechanism of CI remains unclear. Here, we deciphered the dynamic transcriptional changes during the PBS occurrence with and without ethylene pretreatment via time-series transcriptome. We found that ethylene suppressed the cold-signaling gene expression, thereby decreasing the cold sensitivity of the ‘Huangguan’ fruit. Moreover, the “Yellow” module closely correlated with PBS occurrence was identified via weighted gene co-expression network analysis (WGCNA), and this module was related to plant defense via Gene Ontology (GO) enrichment analysis. Local motif enrichment analysis suggested that the “Yellow” module genes were regulated by ERF and WRKY transcription factors. Functional studies demonstrated that PbWRKY31 has a conserved WRKY domain, lacks transactivation activity, and localizes in the nucleus. *PbWRKY31*-overexpressed *Arabidopsis* were hypersensitive to cold, with higher expression levels of cold signaling and defense genes, suggesting that PbWRKY31 participates in regulating plant cold sensitivity. Collectively, our findings provide a comprehensive transcriptional overview of PBS occurrence and elucidate the molecular mechanism by which ethylene reduces the cold sensitivity of ‘Huangguan’ fruit as well as the potential role of PbWRKY31 in this process.

## 1. Introduction

Cold storage is a common and effective way to extend the shelf life and preserve fruit quality. However, some fruits are hypersensitive to cold and susceptible to chilling injury (CI) above freezing, such as banana [1], pineapple [2], litchi [3], and peach [4]. ‘Huangguan’ pear (*Pyrus bretschneideri* Rehd) is a famous early and medium-maturing cultivar widely distributed in North China owing to its appealing appearance and superior edible quality. However, the postharvest ‘Huangguan’ fruit exhibits a cold hypersensitive response, characterized by the occurrence of peel browning spots (PBS) at the early stage of cold storage, resulting in considerable economic loss [5,6].

Previous studies reported that PBS is a symptom of CI, which is related to many physiological processes, such as reactive oxygen species (ROS) metabolism, proline and gibberellin content, the activity of polyphenol oxidase (PPO) and peroxidase (POD), wax composition, and cell lignification [5,6,7,8]. Nevertheless, the transcriptional dynamics during the PBS development and molecular basis of cold hypersensitivity of the ‘Huangguan’ fruit remains unclear.

There are many approaches to reduce the occurrence of PBS in postharvest ‘Huangguan’ fruit, including low-temperature conditioning (LTC), CaCl_2_ dipping, pullulan coating, and ethylene pretreatment [5,6,8,9]. Among them, the effect of ethylene pretreatment is particularly significant, almost completely inhibiting the occurrence of PBS during cold storage, indicating that ethylene can reduce the cold sensitivity of postharvest ‘Huangguan’ fruit [5,6]. However, the molecular mechanism of ethylene reducing the fruit’s susceptibility to cold remains ambiguous.

Over the past two decades, many cold-signaling components have been extensively identified in plants and these molecules can be roughly divided into two pathways: the ICE1-CBF-COR pathway and the CBF-independent pathway [10]. In the ICE1-CBF-COR pathway, CBFs (C-repeat binding factors) are the central regulators of downstream *COR* (*COLD-REGULATED*, *COR*) genes. Upstream of CBFs, ICE1 (inducer of CBF expression 1), CAMTA (Calmodulin-binding transcription activator), OST1 (OPEN STOMATA 1), BZR1/BES1 (brassinazole-resistant 1 and BRI1-EMS-suppressor 1), B1L (BYPASS1-like), ERF105 (Ethylene response factor 105), and SPL9 (Squamosa promoter-binding protein-like 9), etc., are positive regulators of *CBFs*, while CRPK1 (cold-responsive protein kinase 1), MPK3/6 (Mitogen-activated protein kinase 3/6), MYB15 (MYB domain protein 15), ZAT12 (Zinc finger protein 12), and EIN3 (ethylene-insensitive 3), etc., negatively regulate *CBF* expression [11]. Downstream of CBFs, *CORs* are the main molecules conferring cold acclimation in plants [11]. Meanwhile, several molecules have also been identified involving cold signaling in a CBF-independent pathway, including PRO1 (Frostbite1), LOS2 (low expression of osmotically responsive genes 2), ACC1 (Acetyl-COA carboxylase 1), TCF (tolerant to chilling and freezing), BCB (blue copper binding protein), and GI (GIGANTEA), etc. [11]. Moreover, many other cold-induced genes, such as *ATL80* (*Arabidopsis TóxicosenLevadura* 80), *OEP16* (*outer plastid envelope protein 16*), and *LTI6B* (*low temperature-induced protein 6B*) have also been reported [12,13,14].

As a gaseous plant hormone, ethylene regulates a wide range of developmental and physiological processes in plants, especially for climacteric fruit ripening and postharvest storage [15]. Moreover, ethylene is also implicated in regulating the expression of plant cold-signaling genes. In *Arabidopsis*, ethylene reduced cold tolerance by inhibiting the expression of *CBF* and the type-A *Arabidopsis response regulator* (*ARR*) gene [16]. In *SIACS2* (*1-AMINO-CYCLOPROPANE-1-CARBOXYLATE SYNTHASE 2*) antisense tomato, which reduced the endogenous ethylene biosynthesis, the expression of *SICBF1* was lower than in the control fruit, implying that ethylene upregulates *CBF* expression in tomatoes [17]. Likewise, ethylene positively modulates responses to cold stress through the MdERF1B–MdCIbHLH1 cascade in apple, ultimately upregulating *CBF* expression [18]. However, how ethylene regulates the expression of cold-signaling genes in postharvest pear fruit remains poorly understood.

The WRKY genes, belonging to a plant-specific transcription factor family, play a pivotal role in diverse plant biotic and abiotic stresses [19,20]. As a member of WRKY transcription factors, functions of WRKY31 have been studied in some plant species. Overexpression of *PeWRKY31* from *Populus × euramericana* improved plant salt tolerance and insect resistance in transgenic tobacco, indicating that PeWRKY31 is involved in both biotic and abiotic stress [21]. *MdWRKY31* was responsive to drought, cold, salt, and phytohormone abscisic acid (ABA) in apple. Overexpression of *MdWRKY31* in *Arabidopsis* and tobacco enhanced plant sensitivity to ABA [22]. Likewise, the banana *MaWRKY31* was also induced by cold and involved in the ABA-mediated cold tolerance by directly binding to the promoters of ABA biosynthetic genes [1]. As in ‘Nanguo’ pear, *PuWRKY31* was cloned and induced with sucrose. PuWRKY31 bound to the promoters of ethylene biosynthetic genes *PuACS1* and *PuACO1* to promote ethylene production [23]. However, little is known about the role of pear WRKY31 in cold signaling.

In the present study, we investigated the dynamic transcriptional changes during the occurrence of PBS and uncovered the molecular mechanism of ethylene in regulating the cold sensitivity of postharvest ‘Huangguan’ fruit. In addition, we also revealed the role of PbWRKY31 in conferring cold sensitivity.

## 2. Results

### 2.1. Ethylene Pretreatment Reduces Cold Sensitivity of Postharvest ‘Huangguan’ Pear Fruit

**Figure 1:**
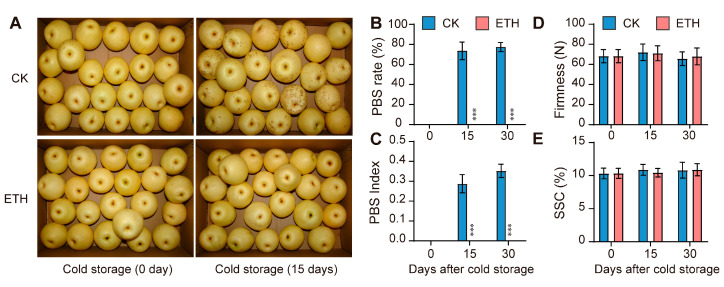
Ethylene pretreatment inhibits peel browning spots (PBS) occurrence. (**A**) Control (CK) and ethylene-pretreated (ETH, 100 μL/L) fruit after 15 days of cold storage (0 °C). (**B**) and (**C**) PBS rate and PBS index were calculated during cold storage. (**D**) and (**E**) Assessment of fruit firmness and soluble solids content (SSC) during cold storage. All data are mean ± standard deviation from three biological replicates. Asterisks indicate significant differences (*** *p* < 0.001) as calculated using two-tailed Student’s *t*-test within each time point.

As Figure 1A shows, the control ‘Huangguan’ pear fruit (CK), which was treated with air, showed a hypersensitive phenotype to cold, specifically characterized by developing peel browning spots (PBS) at the first two weeks of cold storage. On day 15, the PBS rate and PBS index of the CK fruit were 73.6% and 0.29, while on day 30, the PBS rate and PBS index of the CK fruit reached 77.4% and 0.35 (Figure 1A–C). Significantly, ethylene pretreatment (100 μL/L) completely inhibited PBS occurrence (PBS rate and PBS index of ETH samples were zero on day 15 and day 30) but did not change the fruit firmness and soluble solids content after 30 days of cold storage (Figure 1A–E). These results suggested that ethylene pretreatment reduced the cold sensitivity of postharvest ‘Huangguan’ pear fruit.

### 2.2. RNA-seq Experiment Design and Overview

**Figure 2:**
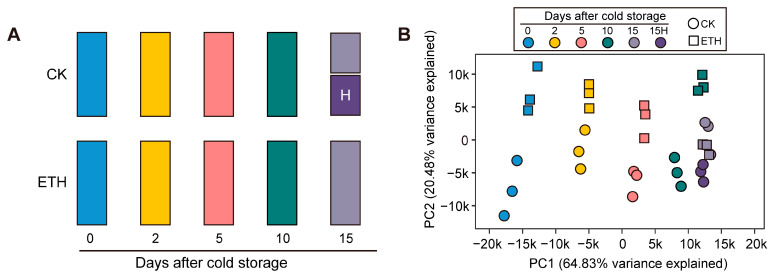
RNA-seq experiment design and principal component analysis (PCA) of sequencing samples. (**A**) The RNA-seq experiment design. For each treatment at each time point, three replicates were set. (**B**) PCA analysis of sequencing samples.

To investigate the molecular mechanism of ethylene pretreatment on the cold sensitivity of ‘Huangguan’ pear fruit, we conducted a time-series RNA-sequencing experiment. As shown in Figure 2A, the peel tissues of both the CK and ETH fruit were collected on days 0, 2, 5, 10, and 15, respectively. For the CK fruit on day 15, the fruit with PBS occurrence was named ‘CK_15d’, while the fruit free of PBS was designated as ‘CK_15dH’ (refers to healthy fruit of the CK group on day 15). For the CK and ETH samples, three replicates were set at each time point (Figure 2A). Thirty-three samples were sequenced, yielding 126 GB of clean reads (Supplementary Table S1). The clean reads were then mapped to the reference genome sequence of *Pyrus bretschneideri* ‘DangshanSuli’ V1.0 [24]. The mapped rates ranged from 78.97% to 79.81% (Supplementary Table S2).

To assess the repeatability and heterogeneity of the sequenced samples, we performed principal component analysis (PCA) and correlation analysis. As shown in Figure 2B, three biological replicates for each sample were clustered together, indicating high reproducibility within biological replicates. Furthermore, the samples were clearly separated by the cold storage time on the PC1 axis, indicating that cold storage time is the key factor responsible for the differences between samples. From the perspective of the PC2 axis, the samples were separated via the CK and ETH treatment, indicating that ethylene pretreatment plays a key role in the heterogeneity between the CK and ETH samples (Figure 2B). Interestingly, the CK_15dH samples were closer to the ETH_15d samples than the CK_15d samples (Figure 2B). As expected, correlation analysis of the samples also exhibited very tight clustering among the three biological replicates, with CK_15dH samples clustering more closely with ETH_15d samples than the CK_15d samples (Supplementary Figure S1). Taken together, these results show that the reproducibility of sequenced samples is reliable and that the gene expression profiles of the CK_15dH samples are similar to the ETH_15d samples.

### 2.3. Differentially Expressed Gene Analysis

**Figure 3:**
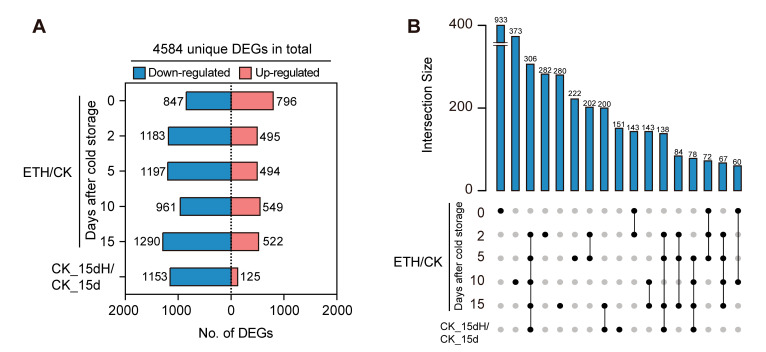
Statistics of differentially expressed genes (DEGs). (**A**) Counts of DEGs for each time point. (**B**) Upset plot illustrating the intersection of DEGs.

To examine the transcriptional differences between CK and ETH group samples, differentially expressed genes (DEGs) at each time point were calculated based on fold change (FC) ≥ 2 and FDR < 0.01. A total of 4584 unique DEGs were identified across all time points between samples from the CK and ETH groups. On day 0, 847 genes were downregulated, and 796 genes were upregulated after ethylene treatment. During cold storage, the number of the downregulated genes was almost double that of the upregulated genes in the ETH samples, indicating that ethylene pretreatment mainly repressed gene expression (Figure 3A).

On day 0, there were 933 specific DEGs, while there were only 306 common DEGs during the cold storage. The numbers of time-specific DEGs on days 2, 5, 10, and 15 were 282, 222, 373, and 280, respectively (Figure 3B). Taken together, this result suggests that DEGs changed during the cold storage, with each time point having its specific DEGs.

### 2.4. Effect of Ethylene Pretreatment on the Expression of Cold-Signaling Genes

**Figure 4:**
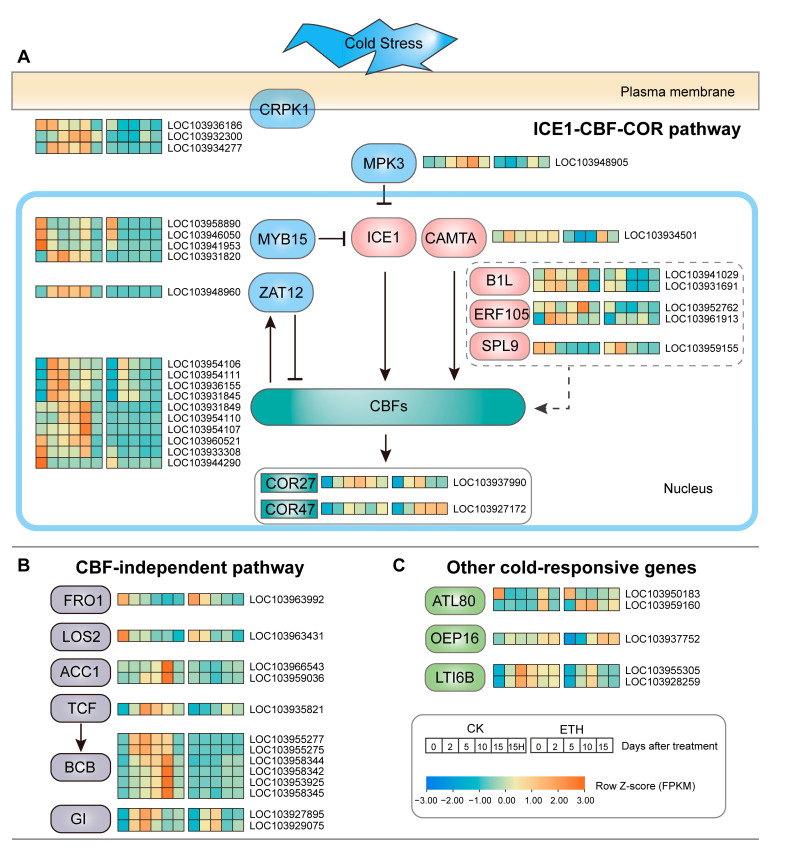
Effect of ethylene on the expression of cold-signaling components during cold storage in ‘Huangguan’ pear. (**A**) ICE1-CBF-COR pathway. The pink ovals and blue ovals represent genes that positively and negatively regulate *CBFs*, respectively. (**B**) CBF-independent pathway. (**C**) Other cold-responsive genes.

Since ethylene pretreatment greatly reduced the cold sensitivity of the ‘Huangguan’ fruit, we then wondered whether the PBS occurrence was correlated with the expression of cold-signaling genes and how ethylene regulates their expression. We selected the cold-signaling genes from the 4584 unique DEGs based on the literature and classified these genes into three categories: the ICE1-CBF-COR pathway, CBF-independent pathway, and other cold-responsive genes (Figure 4). In the ICE1-CBF-COR pathway, the expression peaks of different *CBFs* were different in the CK sample, but they were all suppressed in the ETH samples. Likewise, the expressions of *CRPK1*, *MPK3*, *MYB15*, *CAMTA*, *B1L*, *ERF105*, and *SPL9* were also induced by cold in the CK samples, but significantly repressed in the ETH samples (Figure 4A). Downstream of CBFs, *COR27* was upregulated by cold and inhibited by the ethylene pretreatment. However, the expression of *COR47* was induced by cold in both the CK and ETH samples (Figure 4A).

In the CBF-independent pathway, cold storage induced the expression of *LOS2*, *ACC1*, *TCF*, *BCB*, and *GI*, but their expression levels were dramatically reduced in the ETH samples, more so than in the CK samples. Exceptionally, *FRO1* expression did not change obviously in the CK and ETH samples (Figure 4B).

For other cold-responsive genes, the expression of *OEP16* and *LTI6B* was upregulated by cold but repressed with the ethylene pretreatment. However, one of the *ATL80* (LOC103959160) was induced via the ethylene pretreatment (Figure 4C).

As for the comparison between CK_15d and CK_15dH samples, the expression of most of the cold-signaling genes was lower in the CK_15dH samples than in the CK_15d samples, resembling the expression trend in the ETH_15d samples. This observation suggests that the cold sensitivity of the CK_15dH samples is similar to ETH_15d samples but not CK_15d samples (Figure 4A–C).

To verify the expression profiles of these cold-signaling genes from RNA-seq results, a RT-qPCR was performed. The results showed that *PbCRPK1*, *PbZAT12*, *PbMYB15*, *PbCAMTA*, *PbCBF*, *PbTCF*, and *PbBCB* were all induced by cold, but the amplitude of induction was significantly repressed in the ETH samples. Furthermore, the expression levels of these candidates were lower in the CK_15dH and ETH_15d samples compared with the CK_15d samples, except for *PbCAMTA* (Supplementary Figure S2). Taken together, these results indicated that the expressions of most cold-signaling genes in both CBF-dependent and CBF-independent signaling pathways were higher in the CK samples but repressed in the ETH samples.

### 2.5. Weighted Gene Co-Expression Network Analysis (WGCNA)

**Figure 5:**
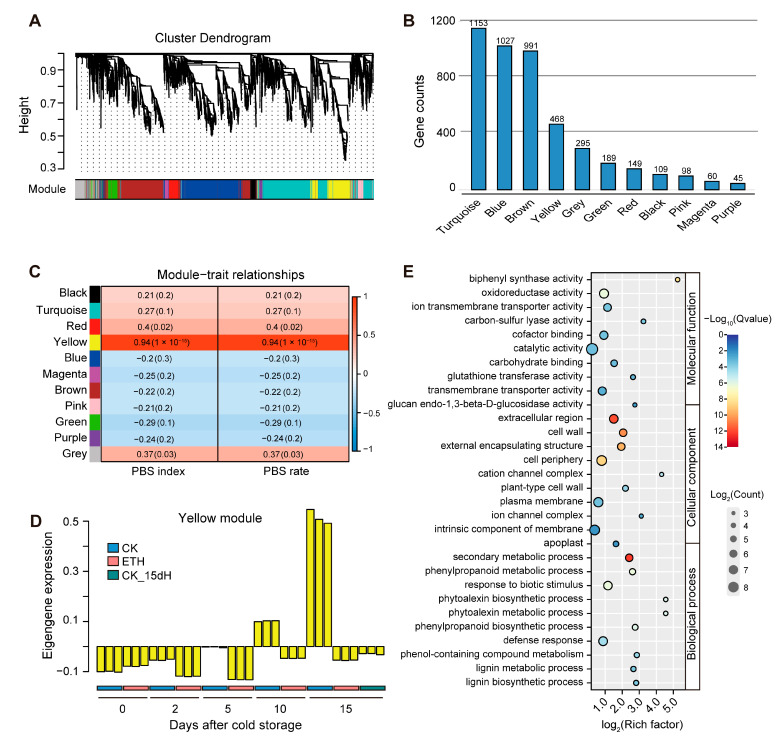
Weighted gene co-expression network analysis (WGCNA) and GO enrichment analysis. (**A**) Construction of WGCNA modules. (**B**) Gene counts in each module. (**C**) Module–trait relationships. Numbers in each grid are Pearson correlation coefficient and significant *p*-value in brackets. (**D**) Eigengene expression in the ‘Yellow’ module. (**E**) GO enrichment analysis of the ‘Yellow’ module.

To screen the potential gene modules closely associated with the PBS occurrence, weighted gene co-expression network analysis (WGCNA) was performed on 4584 unique DEGs and a total of 11 gene modules were identified (Figure 5A,B). The “Turquoise” module was the largest gene module with 1153 genes, followed by the “Blue module (1027 genes)”, “Brown module (991 genes)”, and “Yellow module (468 genes) (Figure 5B).

Module–trait relationships analysis showed that the “Yellow” module was strongly and positively correlated with the PBS index and PBS rate (*r* = 0.94, *p* = 1 × 10^−15^) (Figure 5C). The eigengene expression profile of the “Yellow” module showed that genes in this module were induced by cold, peaked on day 15, and were suppressed in the ETH samples and CK_15dH samples (Figure 5D).

Next, to further explore the significant biological function of the “Yellow” module genes, we performed a Gene Ontology (GO) enrichment analysis. As for the “Molecular function”, the terms “biphenyl synthase activity”, “carbon-sulfur lyase activity”, and “glucan endo-1,3-beta-D-glucosidase activity” were highly enriched. As for the “Cellular component”, the terms “extracellular region”, “cell wall”, and “cation channel complex” were enriched. As for the “Biological process”, “phenylpropanoid metabolic process”, “phytoalexin biosynthetic and metabolic process”, “lignin biosynthetic and metabolic process”, and “defense response” were significantly enriched (Figure 5E). These enriched terms suggest that the “yellow” module genes are closely related to plant defense, suggesting that PBS occurrence may be a manifestation of cold-induced hypersensitive response (HR).

### 2.6. Expression of Defense-Related Genes in the “Yellow” Module

To further investigate the defense-related genes in the ‘Yellow’ module, we categorized these defense-related genes based on their annotation and function. As shown in Table 1, 79 genes were identified as defense-related genes and grouped into 12 categories. Among them, there were 64 genes of “Pathogenesis related proteins”, “Phytoalexins synthesis”, “Lignin synthesis”, and “Glutathione S-transferase”, accounting for 79.7% of total defense-related genes in the “Yellow” module (Table 1).

**Figure 6:**
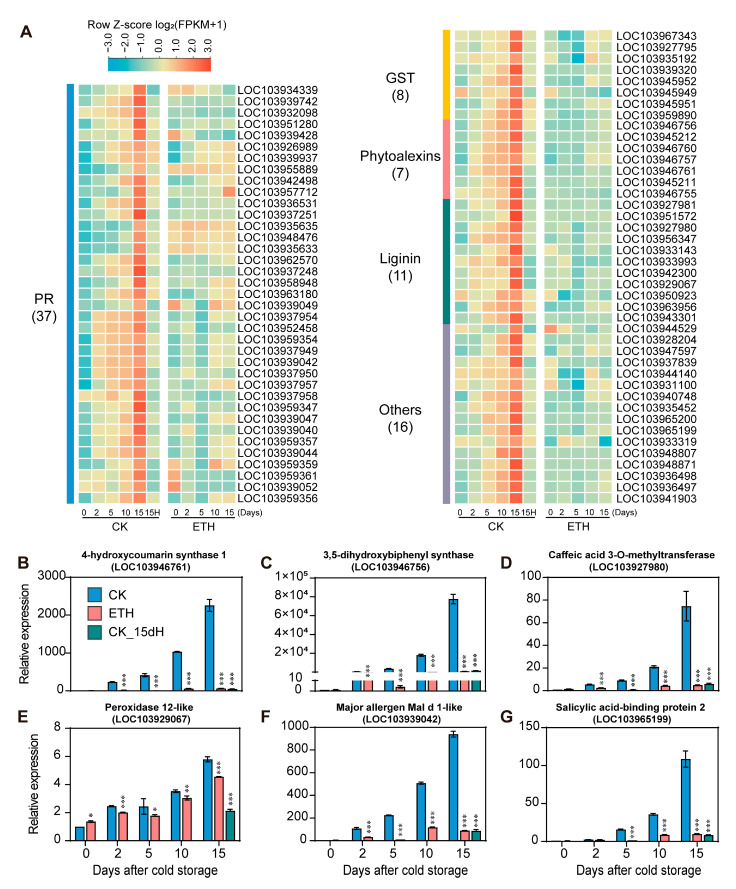
Expression of defense-related genes in the ‘Yellow’ module. (**A**) Heatmap showing the expression of defense-related genes in the ‘Yellow’ module. (**B**–**G**) Validation of defense-related gene expression via a RT-qPCR. All data are mean ± standard deviation from three biological replicates. Asterisks indicate significant differences (* *p* < 0.05, ** *p* < 0.01, *** *p* < 0.001) as calculated using two-tailed Student’s *t*-test within each time point.

The heatmap of gene expression showed that these defense-related genes were induced by cold and suppressed in the ETH and CK_15dH samples, similar to the eigengene expression pattern of the “Yellow” module (Figure 6A). To validate the RNA-seq results, six genes were selected and confirmed via a RT-qPCR (Figure 6B–G). In conclusion, these results suggest that PBS occurrence may be a symptom of a cold-induced hypersensitive response (HR).

### 2.7. Identification of Transcription Factors Regulating Gene Expression of the “Yellow” Module via CentriMo Analysis

**Figure 7:**
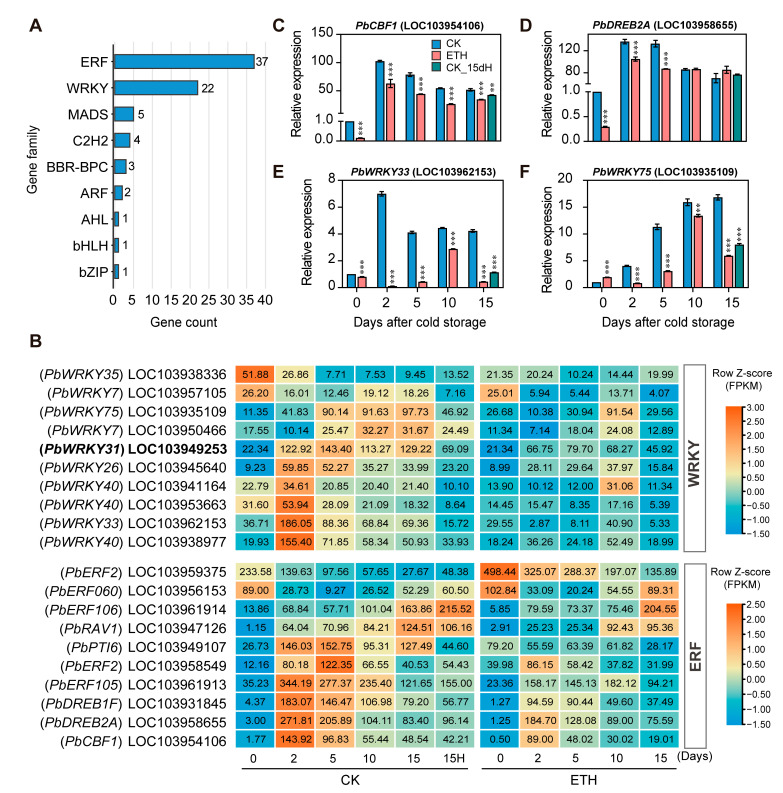
Identification of transcription factors regulating gene expression of the ‘Yellow’ module via CentriMo analysis. (**A**) Counts of different family transcription factors identified via CentriMo analysis. (**B**) Heatmap showing the top 10 expressed transcription factors of the *WRKY* and *ERF* families. (**C**–**F**) Validation of gene expression via a RT-qPCR. All data are mean ± standard deviation from three biological replicates. Asterisks indicate significant differences (** *p* < 0.01, *** *p* < 0.001) as calculated using two-tailed Student’s *t*-test within each time point.

To identify the primary transcription factors (TFs) that regulate gene expression of the “Yellow” module, the promoter sequences of “Yellow” module genes were extracted and submitted to the online tool CentriMo. A total of 76 TFs were identified, belonging to different gene families including *WRKY*, *ERF*, *MADS*, and *C2H2*, etc. (Supplementary Table S4). Among these TFs, there are 59 TFs in *ERF* and *WRKY* families, accounting for 77.6% of the total, suggesting that ERF and WRKY TFs may be involved in regulating gene expression in the “Yellow” module (Figure 7A).

We then found the 37 WRKY and 68 ERF TFs from the 4584 unique DEGs (Supplementary Table S5). The average expression levels of these TFs in all samples were calculated, and the top 10 ERF and WRKY TFs were selected to draw an expression heatmap. As shown in Figure 7B, *PbERF105*, *PbERF106*, *PbRAV1*, *PbPTI6*, *PbERF2*, *PbDREB1F*, *PbDREB2A*, and *PbCBF1* were rapidly induced after two days of cold storage and largely repressed in the ETH samples. Likewise, the expression of *PbWRKY26*, *PbWRKY31*, *PbWRKY33*, *PbWRKY75*, and three *PbWRKY40s* were also rapidly induced by cold but suppressed in the ETH samples. Furthermore, the expressions of two ERF-TFs, *PbCBF1* and *PbDREB2A*, and two WRKY-TFs, *PbWRKY33* and *PbWRKY75*, were verified by via a RT-qPCR (Figure 7C–F). Among these WRKY TFs, *PbWRKY31* showed the highest average expression level (Figure 7B), implying *PbWRKY31* may be involved in regulating the cold sensitivity of fruit.

### 2.8. Sequence Conservation, Gene Expression, Transcriptional Activation, and Subcellular Localization of PbWRKY31

Because *PbWRKY31* was identified as a primary transcription factor regulating gene expression of the “Yellow” module, with the highest expression level among 22 WRKY TFs in Figure 7A,B, we selected *PbWRKY31* for further functional studies.

**Figure 8:**
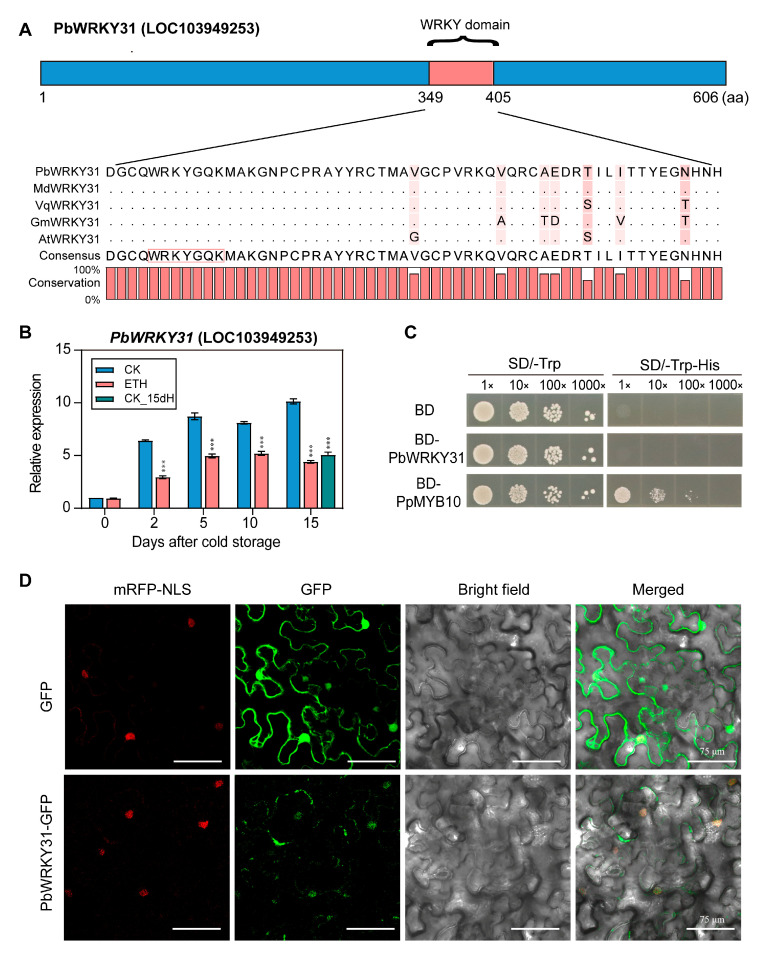
Sequence conservation, gene expression, transcriptional activation, and subcellular localization of PbWRKY31. (**A**) Conserved sequence analysis of the WRKY domain of PbWRKY31 with those of WRKY31 from other species. MdWRKY31 (apple): MD05G1349800; VqWRKY31 (grapevine): VIT_10s0116g01200; GmWRKY31 (soybean): XP_003546160.1; AtWRKY31 (*Arabidopsis*): AT4G22070. The WRKYGQK motif is marked with a red rectangle. (**B**) Validation of *PbWRKY31* expression via a RT-qPCR. All data are mean ± standard deviation from three biological replicates. Asterisks indicate significant differences (*** *p* < 0.001) as calculated using two-tailed Student’s *t*-test within each time point. (**C**) Transcriptional activation of PbWRKY31 in the yeast. The GAL4 DNA-binding domain (**B**,**D**) and BD-PpMYB10 were used as the negative and positive control, respectively. SD/-Trp, synthetic dextrose medium lacking Trp; SD/-Trp-His, synthetic dextrose medium lacking both Trp and His. The transformed yeast cells were diluted at 1, 10, 100, and 1000-fold for dropping on the selective plates. (**D**) Subcellular localization of PbWRKY31 in *Nicotiana benthamiana* leaf cells. Scale bar: 75 μm. mRFP-NLS is a nucleus marker.

To investigate the function of PbWRKY31 in plants, we first aligned the protein sequence of PbWRKY31 with other known WRKY31 protein sequences. As shown in Figure 8A, PbWRKY31 consists of 606 amino acids, and the WRKY domain is highly conserved in pear, apple [22], grapevine [25], soybean [26], and *Arabidopsis* [27], particularly the 7-peptide WRKYGQK motif at the N-terminus [20], implying that they may have relatively conserved functions (Figure 8A).

Next, we confirmed the expression pattern of *PbWRKY31* via a RT-qPCR. As shown in Figure 8B, the overall expression trend of *PbWRKY31* was consistent with the RNA-seq results (Figure 7B and Figure 8B). The magnitudes of cold induction of *PbWRKY31* in the CK_15dH and ETH_15d samples were similar, and both were lower than the expression in the CK_15d samples (Figure 8B). This result suggests the expression level of *PbWRKY31* is positively correlated with the cold sensitivity of ‘Huangguan’ fruit.

To test whether PbWRKY31 has transcriptional activity, a transactivation activity assay was performed in yeast. The coding sequence of *PbWRKY31* was cloned into the pGBKT7 vector fused to the GAL4 DNA-binding domain. The obtained BD-PbWRKY31, negative control (pGBKT7 empty vector), and positive control (BD-PpMYB10) were transformed into yeast strain Y187, respectively. The results showed that the yeast carrying the pGBKT7 empty vector (BD) and BD-PbWRKY31 could not grow on the selective medium (SD/-Trp-His), while the positive control BD-PpMYB10 grew well (Figure 8C). This result indicates that PbWRKY31 lacks transactivation activity and may require other transcriptional regulators to exert its full function in vivo.

Nuclear localization is critical for transcription factor function. The coding sequence of *PbWRKY31* was inserted into the pBI121-GFP vector (p35S-PbWRKY31-GFP). The obtained vector was transformed into Agrobacterium and co-infiltrated into young tobacco leaves together with a nuclear marker (NSL-mCherry). As shown in Figure 8D, the green fluorescence of PbWRKY31-GFP overlapped well with the red nucleus marker, and the extranuclear GFP signal was weak, while free GFP existed both in the nucleus and outside the nucleus (Figure 8D).

### 2.9. Overexpression of PbWRKY31 Increases Cold-Sensitivity in Arabidopsis

**Figure 9:**
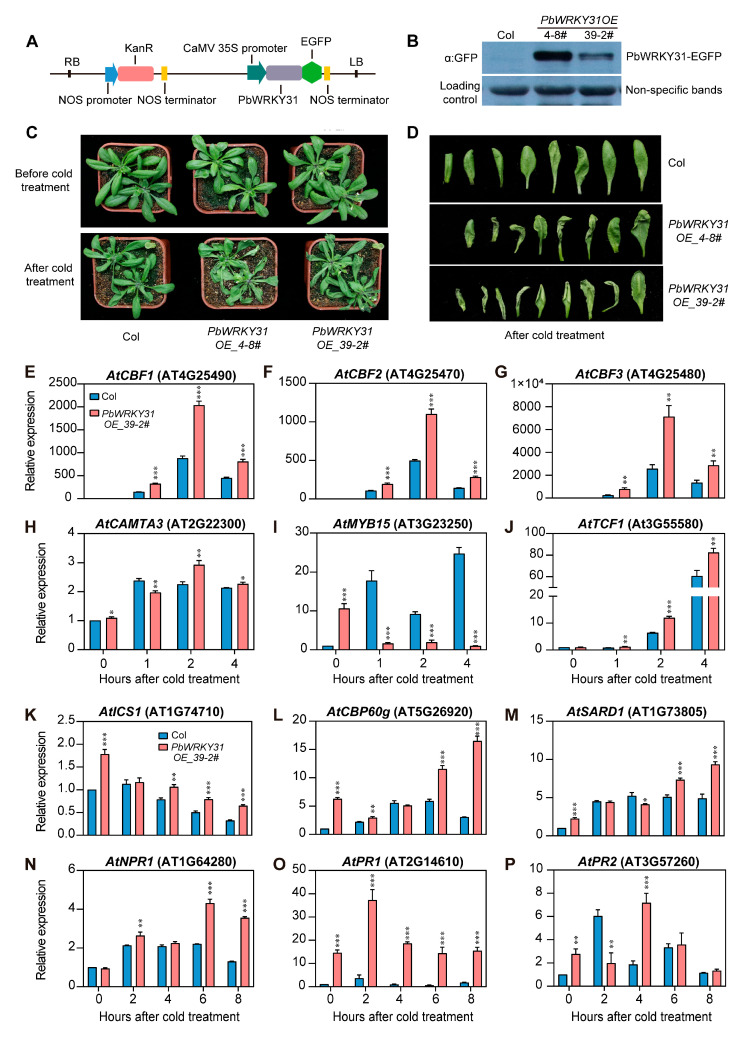
Overexpression of *PbWRKY31* enhances cold sensitivity in *Arabidopsis*. (**A**) Schematic representation of *PbWRKY31* over-expression vector. (**B**) Confirmation of PbWRKY31 over-expression via immunoblotting. (**C**,**D**) *PbWRKY31* overexpressed *Arabidopsis* plants (*PbWRKY31OE*) were hypersensitive to cold treatment (0 °C for 2 days) compared with wild-type control (Col). (**E**–**J**) Expression of cold signaling components in Col and *PbWRKY31OE* plants after cold treatment (4 °C for 0, 1, 2, 4 h). (**K**–**P**) Expression of salicylic acid biosynthetic and signaling components in Col and *PbWRKY31OE* plants after cold treatment (4 °C for 0, 2, 4, 6, and 8 h). All data are mean ± standard deviation from three biological replicates. Asterisks indicate significant differences (* *p* < 0.05, ** *p* < 0.01, *** *p* < 0.001) as calculated using two-tailed Student’s *t*-test within each time point.

To investigate the role of PbWRKY31 in regulating plant cold sensitivity, the p35S-PbWRKY31-GFP vector containing a kanamycin selection marker was transformed into *Arabidopsis* (Figure 9A). Two independent homozygous lines of *PbWRKY31OE_4-8#* and *PbWRKY31OE_39-2#* were confirmed via immunoblotting and used for the phenotypic analysis (Figure 9B).

Three-week-old *PbWRKY31OE* and Col plants were treated at 0 °C ± 0.5 °C for two days. Surprisingly, *PbWRKY31OE_4-8#* and *PbWRKY31OE_39-2#* plants were more sensitive to cold treatment than the Col and exhibited a highly leaf-shrunken phenotype (Figure 9C,D). Consistent with this phenotypic difference, the expression levels of cold-signaling genes, which are homologous to the pear cold-signaling genes mentioned in Figure 4, were significantly higher in the *PbWRKY31OE* seedlings than the Col before or after cold treatment (Figure 9E–J). Taken together, these results suggest that PbWRKY31 increases cold sensitivity in *Arabidopsis*.

Since the “Yellow” module genes are closely related to plant defense (Figure 5E), we then examined the transcript levels of defense-related genes in *Arabidopsis* after cold treatment, especially those related to the central plant defense hormone salicylic acid (SA). In the *PbWRKY31OE* seedlings, the expression levels of key SA biosynthesis gene *AtICS1*, two positive regulators of SA biosynthesis, *AtCBP60g* and *AtSARD1*, key transcription factor *AtNPR1*, and downstream SA marker genes *AtPR1* and *AtPR2* [28] were higher than the Col (Figure 9K–P). These data suggest that PbWRKY31 is involved in cold-induced defense responses.

## 3. Discussion

The occurrence of PBS in postharvest ‘Huangguan’ fruit is considered a symptom of a chilling injury [5,6]. In this study, we investigated the dynamic transcriptional changes in control (CK) and ethylene-pretreated (ETH) fruit during cold storage.

To evaluate the repeatability of the sequenced samples, we performed PCA analysis, and the result showed good repeatability of biological replicates (Figure 2B). Importantly, CK and ETH samples could be separated at each time point on the PC2 axis, indicating that the difference between the CK and ETH samples occurred immediately after ethylene treatment (Figure 2B). Our previous results showed that ethylene treatment promoted endogenous ethylene production in the ‘Huangguan’ pear [6]. Furthermore, the CK_15dH samples without PBS were closer to the ETH_15d samples than the CK_15d samples (Figure 2B). These results suggested that endogenous ethylene is crucial for PBS occurrence. Consistently, this speculation is also supported by the comparison of gene expression in the CK_15dH and ETH_15d samples. Most cold signaling and defense genes were expressed at lower and similar levels in CK_15dH and ETH_15d samples, but higher in CK_15d samples (Figure 4 and Figure 6, and Supplementary Figure S2).

Phytohormones are involved in the regulation of cold signaling in plants, such as auxin, ABA, ethylene, cytokinins, gibberellins, jasmonic acid, and brassinosteroids [29]. Among them, the regulation of ethylene on cold-signaling genes is controversial. In *Arabidopsis* and soybean, ethylene negatively regulates *CBF* expression [16,30]. In contrast, ethylene positively regulated *CBF* in tomato, grapevine, and apple [17,18,31]. Here, we found that ethylene exerted a global repressive effect on the expression of cold-signaling genes in both CBF-dependent and CBF-independent signaling pathways (Figure 4 and Supplementary Figure S2). This may be the explanation for how ethylene reduces the cold sensitivity in postharvest ‘Huangguan’ pears. However, the molecular mechanism by which ethylene negatively regulates numerous cold-signaling genes requires further investigation.

In this study, we performed WGCNA analysis and identified a ‘Yellow’ module closely correlated with the PBS occurrence (Figure 5A–C). Functional GO enrichment analysis showed that this ‘Yellow’ module was involved in plant defense, and many defense-related terms were significantly enriched, such as “biphenyl synthase activity”, “phytoalexin biosynthetic and metabolic process”, “lignin biosynthetic and metabolic process”, and “defense response” (Figure 5E). Further classification of defense-regulated genes in the “Yellow” module showed that 64 genes were related to “Pathogenesis related proteins”, “Phytoalexins synthesis”, “Lignin synthesis”, and “Glutathione S-transferase” (Table 1). In addition, two *SABP2* (*salicylic acid-binding protein 2*) genes that convert methyl salicylate (MeSA) into SA [32] were also highly expressed in the fruit with PBS (CK_15d sample) but repressed in the fruit without PBS (CK_15dH and ETH_15d samples) (Table 1 and Figure 6A,G). It is reported that temperature has a huge impact on plant defense, especially the SA level and signaling [33]. High temperature downregulates SA synthesis by modulating *CBP60g* and *SARD1* to reduce plant immunity [34]. Whereas low-temperature upregulates multiple SA biosynthesis and signaling genes, and ethylene inhibits SA biosynthesis in both normal and low-temperature in *Arabidopsis* [35]. These reports are highly consistent with our data, as most of the defense-related genes, including two *SABP2* and thirty-seven *PR* genes in SA signaling, were upregulated by cold and repressed using the ethylene pretreatment (Figure 6). Taken together, these data suggest that the occurrence of PBS may be a hypersensitivity reaction (HR) induced by cold, possibly through enhanced salicylic acid signaling, and ethylene reduced PBS occurrence by the inhibition of SA’s action.

We also tried to identify the potential transcription factors regulating gene expression in the “Yellow” module via CentriMo analysis. The results implied that ERF and WRKY TFs may be the candidates (Figure 7A). We selected *PbWRKY31* for functional studies due to its high abundance and cold responsiveness (Figure 7B and Figure 8B). PbWRKY31 has a conserved WRKY domain in many species, but it does not have transactivation activity (Figure 8A–C). On the contrary, OsWRKY31 has transactivation activity in yeast, indicating that sequences outside the WRKY domain may also be necessary for its activation activity [36]. Subcellular localization experiments found that PbWRKY31 located in and outside the nucleus, while WRKY31 from *Arabidopsis*, *Populus × euramericana*, rice, and soybean are all exclusively located in the nucleus [21,26,27,36], suggesting PbWRKY31 may also function outside the nucleus.

Further ectopic overexpression of *PbWRKY31* in *Arabidopsis* demonstrated that the *PbWRKY31OE* plants showed a cold hypersensitive phenotype (Figure 9C, D) and higher expressions levels of cold-signaling genes, suggesting that PbWRKY31 confers cold sensitivity in *Arabidopsis* (Figure 9E–J). In the soybean, GmWRKY31 positively regulates *GmNPR1* expression by binding to the W-box of the *GmNPR1* promoter, and overexpression of *GmWRKY31* upregulated the expressions of several *PR* genes [26]. Similarly, overexpression of *VqWRKY31* in grapevine increased levels of salicylic acid and promoted the expression of *PR* genes [25]. In our study, expression of *ICS1*, *CBP60g*, *SARD1, NPR1*, *PR1,* and *PR2* were also elevated in the *PbWRKY31OE* plants. These data suggest that WRKY31 has a conserved role in promoting the expression of SA synthesis genes and downstream *PR* genes. However, whether PbWRKY31 is indispensable for conferring cold sensitivity, and how the endogenous SA level contributes to the PBS occurrence in ‘Huangguan’ fruit during cold storage, require further study.

In conclusion, we determined that dynamic transcriptional changes during PBS development in the ‘Huangguan’ pear, providing new insights into the occurrence of PBS during cold storage. In addition, we elucidated the molecular mechanism by which ethylene reduces the cold sensitivity of ‘Huangguan’ fruit and the role of PbWRKY31 in conferring cold sensitivity.

## 4. Materials and Methods

### 4.1. Plant Materials and Treatments

‘Huangguan’ pear fruit were harvested at the commercial maturity stage (26 August 2016) from an orchard in Jinzhou, Hebei, China, and immediately transported to the laboratory within 2 h. Fruit with uniform size and no damage were selected for ethylene (ETH, 100 μL/L) and control (CK, air) treatment at room temperature (25 °C) for 12 h. Three biological replicates were performed for each treatment. After treatment, the fruit were placed in the paper box and the peel tissues were collected at day 0. Then, the fruit were transferred to a refrigeration house (0 °C ± 0.5 °C). The fruit quality was determined on days 0, 15, and 30, and fruit peel tissues were sampled on days 2, 5, 10, and 15 during cold storage. The peel tissues were quickly frozen in liquid nitrogen and then stored at −80 °C until needed. On day 15, the control fruit were divided into two groups according to the PBS symptom. The fruit with and without PBS were designated as ‘CK_15d’ and ‘CK_15dH’ (here ‘H’ means healthy), respectively, and the ethylene pretreated samples were named ETH_15d. At each time point, 30 fruit from each treatment were used for sampling.

For *Arabidopsis thaliana*, the seeds were sterilized with 70% ethanol and germinated on a half-strength Murashige and Skoog (MS) medium (PhytoTech Labs, Lenexa, KS, USA) with 1% sucrose and 0.75% Agar powder, stratified at 4 °C for two days and then transferred to a greenhouse with the 16 h light/8 h dark cycle at 22 °C with a relative humidity of 60–70%. One week later, the *Arabidopsis* seedlings were either subjected to cold treatment or transplanted into the soil, and the cold-sensitive phenotype of adult plants was observed three weeks later.

For *Nicotiana benthamiana*, the seeds germinated in the soil. One week later, the tobacco seedlings were transferred to pots. Three-week-old tobacco plants were used for infiltration. The growth condition of tobacco is the same as *Arabidopsis*.

### 4.2. Estimation of PBS Rate and PBS Index

The estimation of the PBS rate and PBS index were performed according to the previous report [6]. Additionally, 30 fruits from three biological replicates of each treatment were used for calculating the PBS rate and PBS index on days 15 and 30. The PBS rate was calculated as follows: PBS rate = number of fruits with PBS/ total number of fruits. The PBS index was visually assessed as follows: 0 = 0%, 1 = 0–25%, 2 = 25–50%, 3 = 50–100% of fruit surface area. Additionally, the calculation formula of the PBS index is: PBS index = ∑ (PBS scale × number of fruit at that scale)/(3 × total number of fruit).

### 4.3. Measurement of Fruit Firmness and Soluble Solids Content

Fruit firmness was measured using a fruit hardness tester (GY-4, TuoPu Instruments, Zhejiang, China), which was equipped with a probe of an 8.0-mm diameter. First, the fruit peel was removed and then the penetrometer was pushed into the fruit flesh at a depth of ∼10 mm. Each fruit was measured on two opposite points on the equatorial line of the fruit and the average value is considered as the firmness of that fruit. Fruit firmness was expressed as Newtons (N), and 10 fruit were measured for each replicate.

After measuring the fruit firmness, the juice was extracted with a pipet. Then, the soluble solids content (SSC) was determined with a digital refractometer (PAL-1, Atago, Japan) by measuring the refractive index of the juice extracted from 10 fruit for each replicate.

### 4.4. RNA Extraction, RNA-seq, WGCNA, and Local Motif Enrichment Analysis

Total RNA was extracted from the peel tissue using the RNAprep Pure Plant Kit according to the manufacturer’s instructions (TIANGEN, Beijing, China). RNA quality was monitored on 1% agarose gels, and RNA quantity was determined via the Qubit^®^ RNA Assay Kit in Qubit^®^ 2.0 Flurometer (Life Technologies, San Francisco, CA, USA). A total amount of 1.5 μg of RNA per sample were used as input material for the RNA sample preparations. Sequencing libraries were generated using the NEBNext^®^ Ultra^TM^ RNA Library Prep Kit for Illumina (NEB, Ipswich, MA, USA) following the manufacturer’s recommendations. The library preparations were then sequenced on an Illumina Hiseq 4000 platform (Illumina, San Diego, CA, USA) and 150 bp paired-end reads were generated.

The raw data was filtered using Trimmomatic to produce clean reads [37]. The clean reads were aligned to the *Pyrus x bretschneideri* genome (https://www.ncbi.nlm.nih.gov/genome/12793, accessed on 13 June 2017) by HISAT2 [38]. Fragments per kilobase of transcript per million fragments (FPKM) were used for the evaluation of gene expression.

Edge R was applied for differential expression analysis [39]. After filtration of the low abundant genes (mean FPKM value of three replicates <2 in both CK and ETH samples), the genes with a fold change ≥2 and false discovery rate (FDR) <0.01 were considered as differentially expressed genes (DEGs).

PCA and sample correlation were calculated in R software (version 4.1.1) and plotted by ggplot2 packages in R and TBtools software [40], respectively. GO enrichment analyses were conducted using TBtools [40]. WGCNA was performed using the WGCNA package in R [41].

For local motif enrichment analysis (CentriMo), the promoter sequences (2000 bp upstream of starting codon ATG) of genes from ‘Yellow’ module were extracted using TBtools [40] and subjected to the online tool CentriMo (version 5.1.1. https://CentriMo-suite.org/CentriMo/tools/centrimo, accessed on 7 March 2020). The JASPAR CORE (2018) plants motif database was selected to identify putative transcription factors binding sites.

### 4.5. Reverse Transcription Quantitative Real-Time PCR (RT-qPCR)

Approximately 500 ng of total RNA from each sample was used for first-strand cDNA synthesis using the PrimeScript^TM^ RT Reagent Kit with gDNA Eraser (Takara, Kusatsu, Japan), and quantitative real-time PCR was conducted using the TB Green Premix Ex Taq^TM^ II (Tli RNaseH Plus) kit (Takara, Kusatsu, Japan) on a 7500 Real-Time PCR System (Applied Biosystems, Waltham, MA, USA) according to the manufacturer’s instructions. *PbACTIN7* and *AtActin* were used as the internal controls for the pear tissue and *Arabidopsis* plants, respectively. The relative expression levels were calculated with the 2^−ΔΔCt^ method. Three biological repeats were performed for each sample, and primer sequences are listed in Supplementary Table S3.

### 4.6. Transactivation Activity Assay

Transactivation activity assay was performed as previously described with minor modifications [42]. The BD-PbWRKY31 vector was constructed by inserting the coding sequence of *PbWRKY31* into pNC-GBKT7 using the Nimble Cloning method [43]. Approximately 500 ng of the BD-PbWRKY31 vector was transformed into yeast strain Y187 using the Super Yeast Transformation Kit II (Coolaber, Beijing, China) and screened sequentially on SD/-Trp and SD/-Trp-His plates at 30 °C incubator for three days before photographing. The same amount of the empty pNC-GBKT7 and BD-PpMYB10 vectors were used as the negative and positive controls, respectively.

### 4.7. Subcellular Localization

The coding sequence of *PbWRKY31* was inserted into the pBI121-GFP vector under the 35S promoter using *pEASY*-Basic Seamless Cloning and Assembly Kit (TransGene, Beijing, China). The resulting 35S:PbWRKY31-GFP vector was confirmed via sequencing and transformed into *Agrobacterium tumefaciens* strain EHA105 (weidibio, Shanghai, China). The agrobacteria were then cultured at 28 °C in an incubator overnight, pelleted via centrifugation, and resuspended in the infiltration buffer (10 mM MgCl_2_, 10 mM MES [pH 5.6], 100 µM acetosyringone) with a final optical density at 600 nm (OD600) of 0.5. Bacterial suspensions were then maintained at room temperature for 1 h and then co-infiltrated with the nuclear marker (NSL-mCherry) strain at a ratio of 1:1 (*v*/*v*) into a young *Nicotiana benthamiana* leaf using a 1 mL plastic syringe. After 2 days of infiltration, the fluorescence signal was observed and photographed via laser confocal microscopy (Leica TCS SP8, Wetzlar, Germany). The empty vector pBI121-GFP co-infiltrated with NSL-mCherry was used as a negative control.

### 4.8. Transformation of Arabidopsis and Cold Treatment

The 35S:PbWRKY31-GFP vector was transformed into *Agrobacterium tumefaciens* strain GV3101 (weidibio, Shanghai, China). The agrobacteria were cultured at 28 °C incubator, pelleted via centrifugation, and resuspended in the transformation buffer (5% sucrose, 0.01% silwet) (Coolaber, Beijing, China) with a final optical density at 600 nm (OD600) of 0.5. Bacterial suspensions were then introduced into *Arabidopsis thaliana* plants (Columbia, Col) via the floral dip method. The transformed seedlings were screened on a half-strength MS medium supplied with 50 mg/L of kanamycin. The T3 homozygous lines 4-8# and 39-2# were further confirmed via immunoblotting using a GFP monoclonal antibody (HT801-01, TransGene, Beijing, China).

For the cold-sensitive phenotype, three-week-old *Arabidopsis* plants were transferred into the refrigeration house (0 °C ± 0.5 °C) for 2 days. For the RT-qPCR assay, one-week *Arabidopsis* seedlings were treated at 4 °C incubator for different hours.

### 4.9. Statistical Analysis

The significant differences (* *p* < 0.05, ** *p* < 0.01, and *** *p* < 0.001) were determined with Student’s *t*-test using GraphPad Prism software (version 9.0.0, GraphPad Software, Inc., La Jolla, CA, USA).

## Figures and Tables

**Table 1:** Classification of defense-related genes in the ‘Yellow’ module.

Defense-Related Genes	Gene ID
**Phytoalexins synthesis (7 genes)**	
3,5-dihydroxybiphenyl synthase	LOC103946756; LOC103945212; LOC103946760; LOC103946757
4-hydroxycoumarin synthase	LOC103946761; LOC103945211; LOC103946755
**Lignin synthesis (11 genes)**	
Caffeic acid 3-O-methyltransferase	LOC103927981; LOC103951572; LOC103927980; LOC103956347; LOC103933143
Caffeoyl-CoA O-methyltransferase	LOC103933993
Peroxidase	LOC103942300; LOC103929067; LOC103950923; LOC103963956
Laccase	LOC103943301
**Pathogenesis related proteins (37 genes)**	
PR-1	LOC103934339; LOC103939742
PR-2 (*β*-1,3-Glucanases)	LOC103932098; LOC103951280; LOC103939428 LOC103926989; LOC103939937; LOC103955889 LOC103942498
PR-3 (Endochitinase)	LOC103957712; LOC103936531
PR-5 (Thaumatin-like proteins)	LOC103937251; LOC103935635; LOC103948476 LOC103935633; LOC103962570; LOC103937248 LOC103958948; LOC103963180
PR-10 (Major allergen Mal d 1and Major allergen Pru av 1)	LOC103939049; LOC103937954; LOC103952458 LOC103959354; LOC103937949; LOC103939042 LOC103937950; LOC103959347; LOC103939047 LOC103939040; LOC103959357; LOC103939044 LOC103959359
PR-14 (Major allergen Pru ar 1)	LOC103937957; LOC103937958; LOC103959361 LOC103939052; LOC103959356
**Glutathione S-transferase (8 genes)**	LOC103967343; LOC103927795; LOC103935192 LOC103939320; LOC103945952; LOC103945949 LOC103945951; LOC103959890
**Pathogenesis-related transcriptional activator PTI5 (2 genes)**	LOC103937839; LOC103944140
**Salicylic acid-binding protein 2 (2 genes)**	LOC103965200; LOC103965199
**Detoxification protein (3 genes)**	LOC103944529; LOC103928204; LOC103947597
**Programmed cell death protein (2 genes)**	LOC103931100; LOC103940748
**Sorbitol dehydrogenase (1 gene)**	LOC103933319
**MLO-like protein (2 genes)**	LOC103948807; LOC103948871
**Disease resistance protein RPP5 (1 gene)**	LOC103935452
**alpha-amylase (3 genes)**	LOC103936498; LOC103936497; LOC103941903

## Data Availability

The raw sequence data reported in this paper have been deposited in the Genome Sequence Archive in National Genomics Data Center, China National Center for Bioinformation/Beijing Institute of Genomics, Chinese Academy of Sciences (GSA: CRA009345) that are publicly accessible at https://ngdc.cncb.ac.cn/gsa, accessed on 28 December 2022.

## Supplementary Materials

The supporting information can be downloaded at: https://www.mdpi.com/article/10.3390/ijms24065326/s1.
