# Supplementary material for: Time-Series Transcriptome Analysis Reveals the Molecular Mechanism of Ethylene Reducing Cold Sensitivity of Postharvest ‘Huangguan’ Pear
Source: Int J Mol Sci. 2023 Mar 10;24(6):5326. doi: 10.3390/ijms24065326 (PMC10049683; doi:10.3390/ijms24065326)
Supplement: Supplementary file 1 [file ijms-24-05326-s001.zip › supplementary files/Figure S1.pdf]

Supplemental Figure S1:

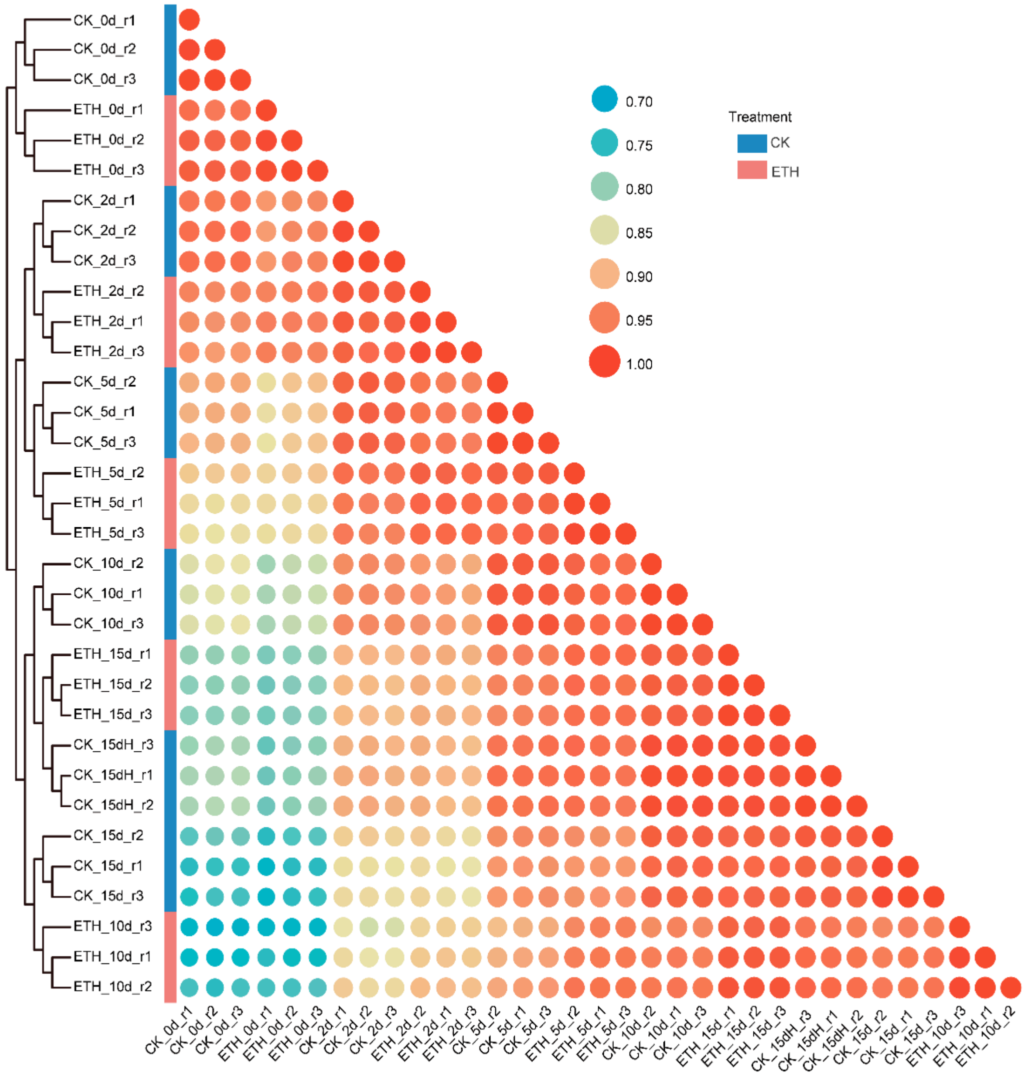

Supplemental Figure S1. Sample correlation analysis. All expressed genes in each sample were used for calculating Pearson's correlation coefficient.
