# Supplementary material for: Time-Series Transcriptome Analysis Reveals the Molecular Mechanism of Ethylene Reducing Cold Sensitivity of Postharvest ‘Huangguan’ Pear
Source: Int J Mol Sci. 2023 Mar 10;24(6):5326. doi: 10.3390/ijms24065326 (PMC10049683; doi:10.3390/ijms24065326)
Supplement: Supplementary file 1 [file ijms-24-05326-s001.zip › supplementary files/Figure S2.pdf]

Supplemental Figure S2:

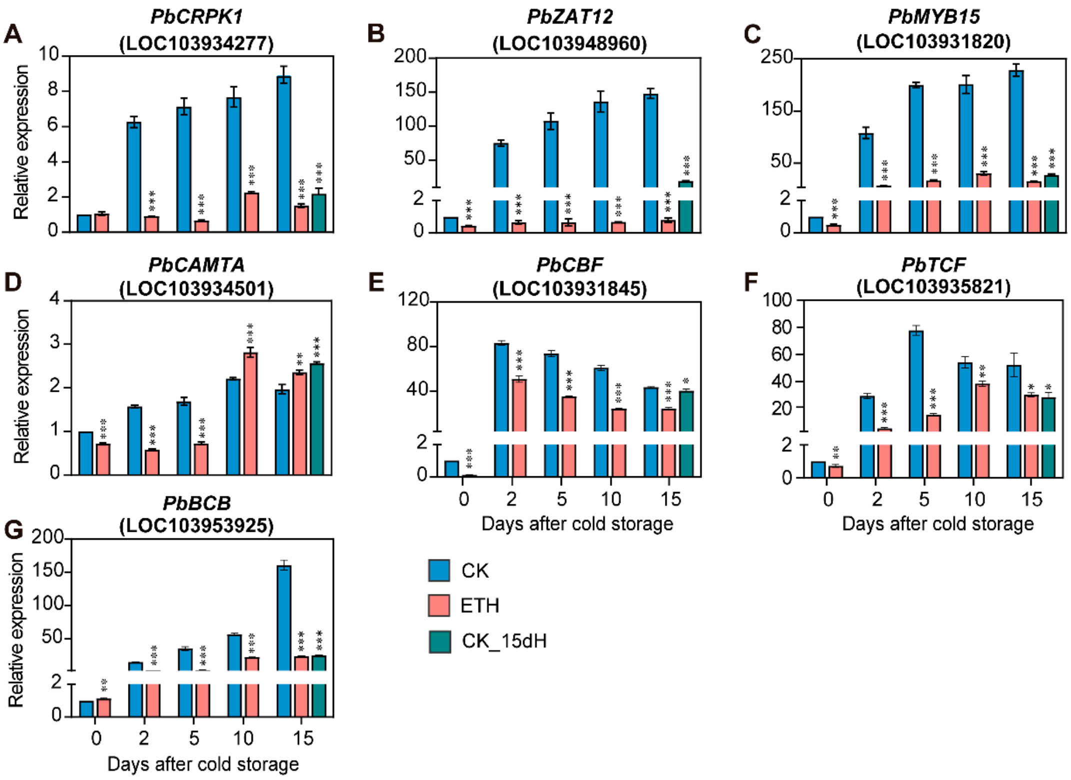

Supplemental Figure S2. Validating the expression of cold-signaling genes by RT-qPCR. All data are mean ± standard deviation from three biological replicates. Asterisks indicate significant differences (*$p < 0.05$, **$p < 0.01$, ***$p < 0.001$) compared with the CK samples as calculated by two-tailed Student's *t*-test within each time point.
