# Supplementary material for: Time-Series Transcriptome Analysis Reveals the Molecular Mechanism of Ethylene Reducing Cold Sensitivity of Postharvest ‘Huangguan’ Pear
Source: Int J Mol Sci. 2023 Mar 10;24(6):5326. doi: 10.3390/ijms24065326 (PMC10049683; doi:10.3390/ijms24065326)
Supplement: Supplementary file 1 [file ijms-24-05326-s001.zip › supplementary files/Table S1. Sequencing data statistics.pdf]

Supplemental Table S1. Sequencing data statistics.

Sample	Raw Reads	Clean Reads	GC (%)	Q30
CK_0d_r1_Read1	21692126	18405399	46.60%	94.58%
CK_0d_r1_Read2	21692126	18405399	46.84%	82.49%
CK_0d_r2_Read1	24325835	20746152	46.54%	93.98%
CK_0d_r2_Read2	24325835	20746152	47.16%	80.24%
CK_0d_r3_Read1	24824145	21700652	46.49%	94.44%
CK_0d_r3_Read2	24824145	21700652	46.94%	82.46%
CK_2d_r1_Read1	21908649	18981995	46.38%	94.11%
CK_2d_r1_Read2	21908649	18981995	46.84%	82.09%
CK_2d_r2_Read1	21905363	18376795	46.27%	93.88%
CK_2d_r2_Read2	21905363	18376795	46.97%	79.21%
CK_2d_r3_Read1	20325517	17292434	46.35%	94.26%
CK_2d_r3_Read2	20325517	17292434	46.90%	80.47%
CK_5d_r1_Read1	21940361	18726536	46.62%	93.99%
CK_5d_r1_Read2	21940361	18726536	47.23%	80.31%
CK_5d_r2_Read1	22696753	19540302	46.48%	94.22%
CK_5d_r2_Read2	22696753	19540302	47.00%	81.43%
CK_5d_r3_Read1	21327637	18383367	46.60%	94.14%
CK_5d_r3_Read2	21327637	18383367	47.10%	81.64%
CK_10d_r1_Read1	21428145	18337956	46.49%	93.92%
CK_10d_r1_Read2	21428145	18337956	47.09%	80.53%
CK_10d_r2_Read1	21659394	19165409	46.16%	94.20%
CK_10d_r2_Read2	21659394	19165409	46.21%	86.42%
CK_10d_r3_Read1	21409975	18512241	46.46%	94.06%
CK_10d_r3_Read2	21409975	18512241	46.94%	82.13%
CK_15d_r1_Read1	22491151	19276689	46.44%	93.99%
CK_15d_r1_Read2	22491151	19276689	47.03%	80.64%
CK_15d_r2_Read1	21778494	18848284	46.24%	94.34%
CK_15d_r2_Read2	21778494	18848284	46.71%	81.72%
CK_15d_r3_Read1	23524655	19988736	46.40%	94.23%
CK_15d_r3_Read2	23524655	19988736	46.95%	80.43%
CK_15dH_r1_Read1	24706435	20917732	46.38%	93.94%
CK_15dH_r1_Read2	24706435	20917732	47.03%	79.80%
CK_15dH_r2_Read1	22790122	19758622	46.26%	94.40%
CK_15dH_r2_Read2	22790122	19758622	46.74%	81.90%
CK_15dH_r3_Read1	21338251	17297735	46.48%	94.13%
CK_15dH_r3_Read2	21338251	17297735	47.25%	77.12%
ETH_0d_r1_Read1	23397426	20152798	46.60%	94.11%
ETH_0d_r1_Read2	23397426	20152798	47.17%	80.98%
ETH_0d_r2_Read1	24270905	20861690	46.51%	94.08%
ETH_0d_r2_Read2	24270905	20861690	47.08%	80.83%
ETH_0d_r3_Read1	20113958	17984484	46.48%	94.23%

ETH_0d_r3_Read2	20113958	17984484	46.53%	85.47%
ETH_2d_r1_Read1	24127221	20626980	46.62%	93.95%
ETH_2d_r1_Read2	24127221	20626980	47.22%	80.45%
ETH_2d_r2_Read1	24408887	20947055	46.54%	93.89%
ETH_2d_r2_Read2	24408887	20947055	47.12%	80.83%
ETH_2d_r3_Read1	22705740	19277008	46.56%	94.12%
ETH_2d_r3_Read2	22705740	19277008	47.12%	80.30%
ETH_5d_r1_Read1	21804102	18690277	46.69%	94.04%
ETH_5d_r1_Read2	21804102	18690277	47.29%	80.66%
ETH_5d_r2_Read1	21379690	18504196	46.56%	94.24%
ETH_5d_r2_Read2	21379690	18504196	47.04%	81.86%
ETH_5d_r3_Read1	27632465	23496243	46.82%	94.05%
ETH_5d_r3_Read2	27632465	23496243	47.39%	80.49%
ETH_10d_r1_Read1	24265440	20665606	46.31%	93.95%
ETH_10d_r1_Read2	24265440	20665606	46.93%	80.19%
ETH_10d_r2_Read1	27273130	23577206	46.26%	94.33%
ETH_10d_r2_Read2	27273130	23577206	46.75%	81.77%
ETH_10d_r3_Read1	22401427	19126459	46.41%	94.15%
ETH_10d_r3_Read2	22401427	19126459	46.92%	80.83%
ETH_15d_r1_Read1	23657571	20143786	46.46%	93.97%
ETH_15d_r1_Read2	23657571	20143786	47.07%	80.18%
ETH_15d_r2_Read1	25017012	21580735	46.33%	94.37%
ETH_15d_r2_Read2	25017012	21580735	46.82%	81.53%
ETH_15d_r3_Read1	25132167	21655782	46.44%	94.26%
ETH_15d_r3_Read2	25132167	21655782	46.93%	81.51%
