# Supplementary material for: Time-Series Transcriptome Analysis Reveals the Molecular Mechanism of Ethylene Reducing Cold Sensitivity of Postharvest ‘Huangguan’ Pear
Source: Int J Mol Sci. 2023 Mar 10;24(6):5326. doi: 10.3390/ijms24065326 (PMC10049683; doi:10.3390/ijms24065326)
Supplement: Supplementary file 1 [file ijms-24-05326-s001.zip › supplementary files/Table S2. Alignment results.pdf]

Supplemental Table S2. Alignment results.

Sample	Total Reads	Unmapped Reads	Mapped Rate	Uniq Mapped Rate
CK_0d_r1	40302349	8230486	79.58%	63.74%
CK_0d_r2	45356580	9318519	79.45%	63.91%
CK_0d_r3	47576055	9736392	79.54%	63.57%
CK_2d_r1	41419501	8571184	79.31%	63.99%
CK_2d_r2	40068103	8386492	79.07%	63.90%
CK_2d_r3	37704085	7736416	79.48%	64.28%
CK_5d_r1	40800039	8508866	79.14%	64.09%
CK_5d_r2	42588571	8858079	79.20%	64.10%
CK_5d_r3	40038447	8290242	79.29%	64.29%
CK_10d_r1	39982187	8208012	79.47%	64.29%
CK_10d_r2	41783801	8713546	79.15%	64.00%
CK_10d_r3	40299920	8337110	79.31%	64.38%
CK_15d_r1	41994246	8669021	79.36%	64.30%
CK_15d_r2	41037969	8629177	78.97%	63.98%
CK_15d_r3	43526870	9052974	79.20%	64.19%
CK_15dH_r1	45594542	9508483	79.15%	64.03%
CK_15dH_r2	43096445	8868696	79.42%	64.18%
CK_15dH_r3	37634975	7732300	79.45%	64.57%
ETH_0d_r1	44022595	8952963	79.66%	64.12%
ETH_0d_r2	45551931	9272570	79.64%	64.18%
ETH_0d_r3	39451325	8076570	79.53%	63.43%
ETH_2d_r1	44993761	9174681	79.61%	64.36%
ETH_2d_r2	45761882	9597081	79.03%	63.58%
ETH_2d_r3	42029142	8485977	79.81%	64.56%
ETH_5d_r1	40780990	8291977	79.67%	64.34%
ETH_5d_r2	40379096	8318987	79.40%	64.07%
ETH_5d_r3	51318177	10494986	79.55%	64.10%
ETH_10d_r1	45164012	9480018	79.01%	63.48%
ETH_10d_r2	51556952	10752031	79.15%	63.52%
ETH_10d_r3	41735764	8748370	79.04%	63.76%
ETH_15d_r1	43993173	9069858	79.38%	63.93%
ETH_15d_r2	47170346	9860717	79.10%	63.56%
ETH_15d_r3	47317663	9786860	79.32%	63.81%
