# Supplementary material for: Time-Series Transcriptome Analysis Reveals the Molecular Mechanism of Ethylene Reducing Cold Sensitivity of Postharvest ‘Huangguan’ Pear
Source: Int J Mol Sci. 2023 Mar 10;24(6):5326. doi: 10.3390/ijms24065326 (PMC10049683; doi:10.3390/ijms24065326)
Supplement: Supplementary file 1 [file ijms-24-05326-s001.zip › supplementary files/Table S3. Primers used in this study.pdf]

Supplemental Table S3. Primers used in this study.

**Primers for DNA constructs**		
Primer Name	Forward (5'-3')	Reverse (5'-3')
BD-PbWRKY31	agtggtctctgtccagtcctATGGACAAAGGATGGGGGCTT	ggtctcagcagaccacaagtATTTCCCGGGAAGCTGCTAATG
GFP-PbWRKY31	agaacacgggggactctagaATGGACAAAGGATGGGGGCTT	agggactgaccacccgggCTATTTCCCGGGAAGCTGCTAATG
**Primers for RT-qPCR (For pear)**		
Gene Name	Forward (5'-3')	Reverse (5'-3')
PbCRPK1 (LOC103934277)	GTTTCTGTTCTGCAGAGGGAT	AGGCCGACTAACAAATGGAGC
PbZAT12 (LOC103948960)	CCTAATCGACCGTCAGCCAT	CACACGTCTTGCACACGAAA
PbMYB15 (LOC103931820)	AGCAAGCTGGGTTGTTGAGA	ATCGGTGCGTCCTGGTAATC
PbCAMTA (LOC103934501)	TCACCGTACGTATCCTCTTGT	TCCGGCGCAACTGTGTAAAT
PbCBF (LOC103931845)	ACATGCCAAGGTTGCTGGAT	CCAGTTCATGTAGCCCCCTG
PbCBF (LOC103954106)	TTCGTCCGTTGCCCAAAAAGA	AGACTAAAATGTAACGGAAACCGTC
PbTCF (LOC103935821)	GAGTTCTCCCAGTCCAAGGG	TTACGCCAATCGTGGAGGAC
PbBCB (LOC103953925)	TACATCTGCACCTTCGCTGG	GAGGGGGAGGAGAGGTAGTG
PbDREB2A (LOC103958655)	TGAAGCAAGAGGAGGGGAGT	ACGTCTCCGGATTGCATTGT
PbWRKY31 *(LOC103949253）*	CTACCTCCTCAACAAGCCGC	CGGGAACATCCTTTCCCCAC
PbWRKY33 (LOC103962153)	ATGTGTCCATGGCTGTGAGG	CGGTTGGCTTTCAGTGGTTG
PbWRKY75 (LOC103935109)	GCACGGTGCAGAGAGACATAA	TTTTGTGACCAAGCCATGAAGT
LOC103946761	CATGGGAGCTCCATCTGTCC	CACGGCTGTCTCGACTGTAA
LOC103946756	GGAATCGGACCAGGACTCAC	ACCGGTTGTGTCATACTCTCG
LOC103927980	AGACAGCTTAGCTAGATGCAC	GCACTGAAGAAACCACCAGC
LOC103929067	CTACTTGGCCTCTGTGGTGG	AAGAGCTCGTTATTACACACATTTT
LOC103939042	CTAAGTTGGTGGCTTCCGGT	GCCAGCCTTAACATGTTCTTCC
LOC103965199	TGTTCGCTCATTGGCAGAGT	ATGGCAAGGGCCAAGTTCAT
PbACTIN7	GGACATTCAACCCCTCGTCT	ATCCTTCTGACCCATACCAACC

**Primers for RT-qPCR (For *Arabidopsis*)**		
AtCBF1 (AT4G25490)	GGAGACAATGTTTGGGATGC	TTAGTAACTCCAAAGCGACACG
AtCBF2 (AT4G25470)	TGACGTGTCCTTATGGAGCTA	CTGCACTCAAAAACATTTGCA
AtCBF3 (AT4G25480)	GATGACGACGTATCGTTATGGA	TACACTCGTTTCTCAGTTTTACAAAC
AtCAMTA3 (AT2G22300)	AATTCGAGTATAAGGTTGCCGA	TTTCAGATTTCGAGCACAACAG
AtMYB15 (AT3G23250)	GCCTGATATTAAACGTGGCAAT	CGAGTCTCTTCTTCAAGTGAGT
AtTCF1 (At3g55580)	ATTCCTCCGGCAGTCGAAAG	CTGGAGTCTCCCCGTGTTTC
AtICS1 (AT1G74710)	GAATTTGCAGTCGGGATCAG	AATTAATCGCCTGTAGAGATGTTG
AtCBP60g (AT5G26920)	CGACTTTCAAGGAAGTTTGG	ACTTTTTGTCGATATGAAACC
AtSARD1 (AT1G73805)	CCTCAACCAGCCCTACGTTA	TAGTGGCTCGCAGCATATTG
AtNPR1 (AT1G64280)	TTTGGAAGGTAGAACCGCAC	ACATTCAACCGCCATAGTGG
AtPR1 (AT2G14610)	GCAACTGCAGACTCATACAC	GTTGTAGTTAGCCTTCTCGC
AtPR2 (AT3G57260)	AGGTTCAGGGATGAGTATAAGA	AGAGATTCACGAGCAAGGGA
AtActin	ATGACTCAGATCATGTTTGAGACC	TCAGTAAGGTCACGACCAGCAA
